# Imaging of Oral SCC Cells by Raman Micro-Spectroscopy Technique

**DOI:** 10.3390/molecules26123640

**Published:** 2021-06-15

**Authors:** Hidetaka Kinoshita, Norio Miyoshi, Toshiyuki Ogasawara

**Affiliations:** 1 Division of Dentistry and Oral Surgery, Fukui General Hospital, Egami, Fukui 910-8561, Japan; toshi@funabashi-dental.com; 2 Department of Rehabilitation Medicine, Fukui College of Health Sciences, Egami, Fukui 910-3190, Japan; 3 Department of Gastroenterology, Faculty of Medicine, Tsukuba University, Tennoudai, Tsukuba 305-8575, Japan; Nmiyoshi@u-fukui.ac.jp

**Keywords:** Raman micro-spectroscopy technique, squamous cell carcinoma (SCC), mapping images

## Abstract

We used Raman micro-spectroscopy technique to analyze the molecular changes associated with oral squamous cell carcinoma (SCC) cells in the form of frozen tissue. Previously, Raman micro-spectroscopy technique on human tissue was mainly based on spectral analysis, but we worked on imaging of molecular structure. In this study, we evaluated the distribution of four components at the cell level (about 10 μm) to describe the changes in protein and molecular structures of protein belonging to malignant tissue. We analyzed ten oral SCC samples of five patients without special pretreatments of the use of formaldehyde. We obtained cell level images of the oral SCC cells at various components (peak at 935 cm^−1^: proline and valine, 1004 cm^−1^: phenylalanine, 1223 cm^−1^: nucleic acids, and 1650 cm^−1^: amide I). These mapping images of SCC cells showed the distribution of nucleic acids in the nuclear areas; meanwhile, proline and valine, phenylalanine, and amide I were detected in the cytoplasm areas of the SCC cells. Furthermore, the peak of amide I in the cancer area shifts to the higher wavenumber side, which indicates the α-helix component may decrease in its relative amounts of protein in the β-sheet or random coil conformation. Imaging of SCC cells with Raman micro-spectroscopy technique indicated that such a new observation of cancer cells is useful for analyzing the detailed distribution of various molecular conformation within SCC cells.

## 1. Introduction

Oral squamous cell carcinoma (SCC) is one of the most common cancers in the craniocervical area, and SCC accounts for approximately 90% of all cancers in the oral cavity [1]. Early detection and suitable treatment influence survival rates, but it is important to obtain an accurate diagnosis to ensure a fine convalescence. Currently, pathological diagnosis is used as the main diagnostic method for oral SCC. 

Raman micro-spectroscopy technique is a vibrational spectroscopy technique that provide information on the molecular conformation of the sample under investigation. Advantages of Raman micro-spectroscopy technique is a direct technique (label-free), in which there is no the need to mark the analytes with other molecules (like Fluorescence spectroscopy, for example); moreover, Raman micro-spectroscopy technique is a non-destructive technique and with the appropriate tools non-invasive for human or biological samples in general [2]. When Raman spectroscopy is applied on biological samples, this method can be used as early disease diagnosis technique. Raman spectra are commonly referred to as molecular fingerprints; molecular vibrations of the sample under investigation appear as a convolution of peaks and bands. Therefore, Raman micro-spectroscopy has been applied as a diagnostic and analytical tool for various cancers [3,4,5,6]. Moreover, it is known that the presence of a molecule to which a spectrum is assigned can be assayed by calculating the peak area of the spectrum [7,8]. Furthermore, it is possible to obtain the mapping images by measuring with a lattice in the selected area, and this mapping image reflects the distribution of molecules assigned to individual peaks on the spectra [9,10]. These mapping images show a minute area, for example a square 60 μm on each side.

In other malignant tissue studies with Raman spectroscopy, the peaks of phospholipids (1079 cm^−1^, 1302 cm^−1^, and 1445 cm^−1^), cellular nucleic acids (1223 cm^−1^ and 1335 cm^−1^), amide III (1265 cm^−1^), CH2 bending mode of proteins and lipids (1436 cm^−1^), and amide I (1650 cm^−1^) were consistently observed [11,12,13]. The focus of these studies was to compare the peak position and intensity on normal tissue and malignant tissue. In our study, we adopted the bands of proline and valine (935 cm^−1^), phenylalanine (1004 cm^−1^), nucleic acids (1223 cm^−1^), and amide I (1650 cm^−1^) and could identify the forms of SCC cells on mapping images based on the distribution of those components. The analysis area of Raman micro-spectroscopy technique was confirmed histopathological and morphological appearance by a hematoxylin-eosin-stained image at both the cell level and the tissue level order.

To date, several studies using the Raman spectra of malignant tissue and normal tissue for various organs have been reported [3,4,5,6,12,13,14]. In our investigation of the Raman spectra of oral SCC cells, we could detect and make imaging of some components using ordinary microscopic sections. Common pathological evaluation is based primarily on morphological appearance rather than biochemistry. Raman spectra are recognized biochemical fingerprints, and cell level imaging has the advantage of both biological and morphological appearance. For the first time, we have successfully obtained amide I (which were missing in the H&E-staining image by de-paraffinization) mapping image of oral SCC cells without special pretreatment use of formaldehyde.

## 2. Results

### 2.1. Oral SCC Tissue and Raman Spectra in Analysis Region

**Figure 1:**
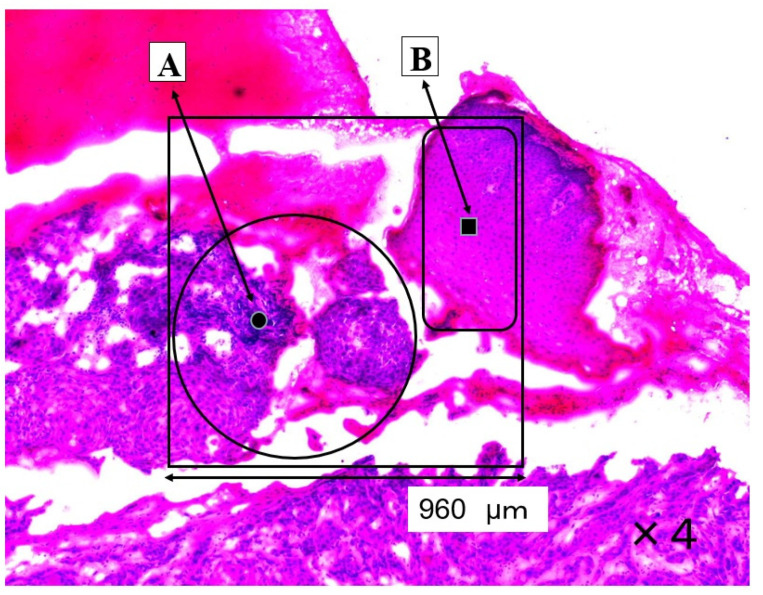
This section shows a SCC region (**A**) and a normal region (**B**). The four-sided area (960 × 960 μm) was analyzed by Raman micro-spectroscopy technique as an SCC area (**A**) and as a normal area (**B**).

The SCC (A) and normal regions (B) are shown in Figure 1. The large ratios of nucleic acid against cytoplasm are concentrated in the cancerous region (A). The pathological diagnosis was oral high differentiation SCC. In contrast, the normal epithelium is shown in region B.

**Figure 2:**
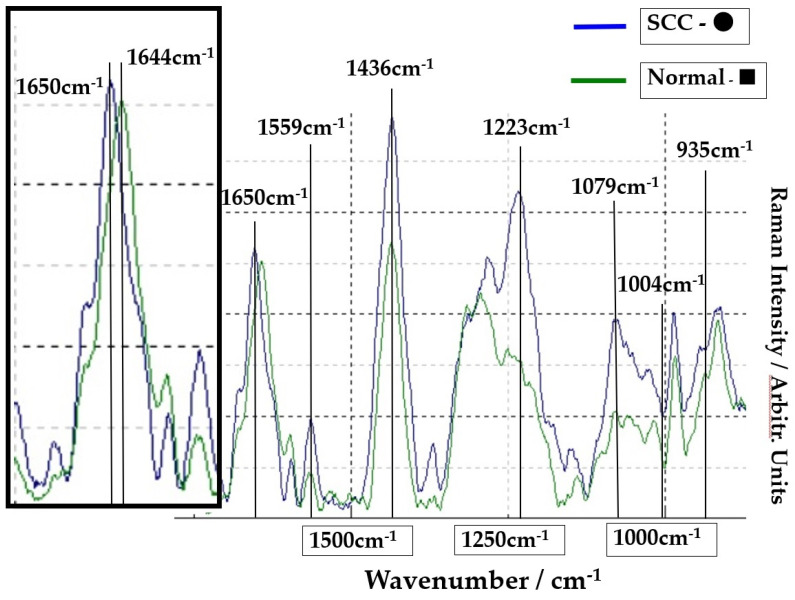
Comparison of spectra at point ● in SCC area (blue line) and at point ■ in normal area (green line). The left frame is the expansion of the peak of 1650 cm^−1^.

The Raman spectra of the SCC region A (point ●) and normal region B (point ■) on Figure 1 are shown in Figure 2. The lower spectra indicate the 800–1800 cm^−1^ region, and the left side spectra are an expanded image of the 1650cm^−1^ region. In Figure 2, the blue line is the spectrum of the SCC region A (point ●), and the green line is the spectrum of the normal region B (point ■). The sharp peaks at 1650 cm^−1^ (amide I) and 1436 cm^−1^ (CH2 bending mode of proteins and lipids) were confirmed in the malignant and normal regions. The amide I peak shifts at 1644 cm^−1^ in the normal region.

**Figure 3:**
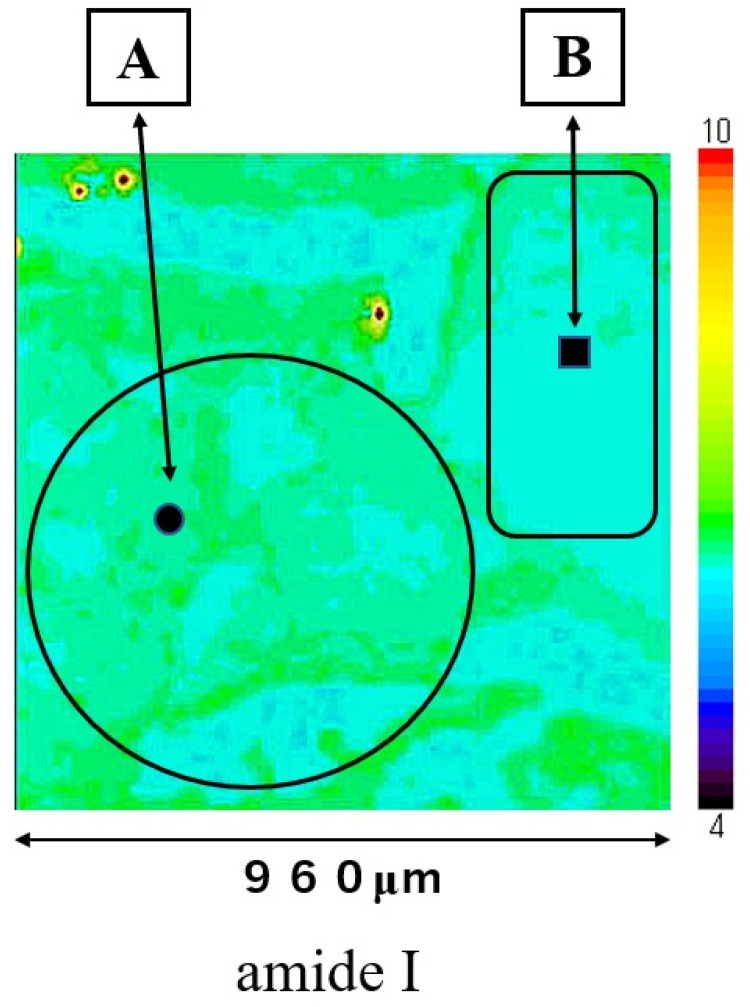
Mapping image of amide I is shown in the square area (960 × 960 μm) on Figure 1. Region (**A**) is SCC and region (**B**) is normal epithelial tissue. Spectra at point ● in SCC area and at point ■ in normal area were shown in Figure 2.

Mapping image of amide I is shown in Figure 3; this measurement area is the square area (960 × 960 μm) on Figure 1. The distribution of amide I was consistent with the extrastructure of SCC and normal regions in Figure 1. The distribution of amide I was lower in normal epithelial region B than in SCC region A. Mapping image of other components could not show a clear distribution compared to that of amide I.

### 2.2. Oral SCC Cells and Raman Spectra of Nuclei and Cytoplasm

**Figure 4:**
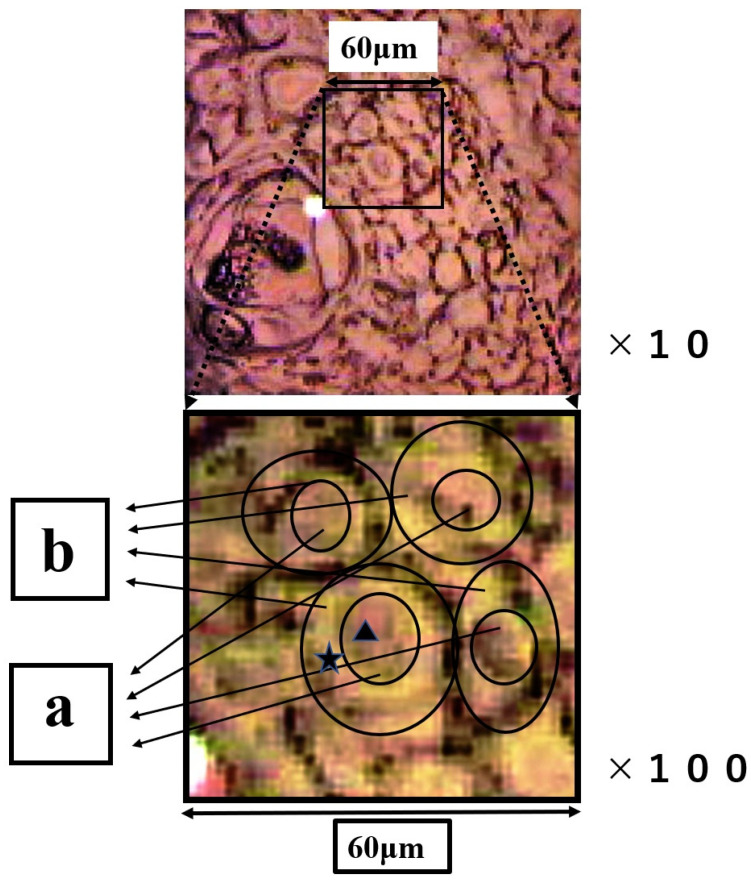
The upper image is the CCD camera (equipped with Raman micro-spectroscope) image on the gold-covered slide glass. The images below are the enlarged images of the four-sided area. In the figure below, the outline of the nucleus and cytoplasm is illustrated, and ▲ and ★ indicate the nucleus and ★ the cytoplasm individually. Region (**a**) is nucleus and region (**b**) is cytoplasm.

**Figure 5:**
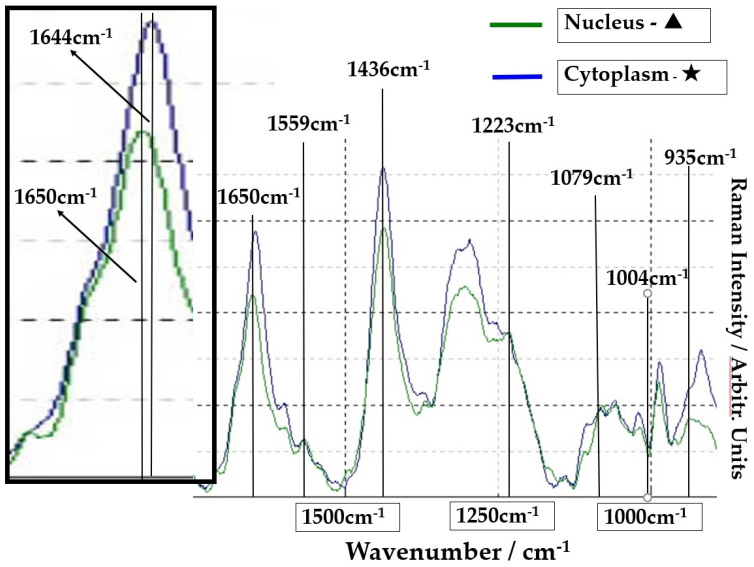
Comparison of spectra from nuclei (point ▲) of SCC cells (green line) and cytoplasm (point ★) (blue line). The left frame is the expansion of the peak of 1650 cm^−1^.

The group of SCC cells with large nuclei is shown in Figure 4. The upper image is the CCD camera (equipped with Raman micro-spectroscope) image of the sample on the slide glass covered with gold, and the lower images are enlarged. The square (60 × 60 μm) in the lower images contains four SCC cells in the center. These lower two images are the same, and the left image shows the outline of nucleus and cytoplasm. This four-sided area of the lower image was analyzed by Raman micro-spectroscope technique. Four points ‘a’ are located in the nuclei, and cytoplasm (b) and cell wall are confirmed around these nuclei (a). The Raman spectra of nucleus (point ▲) and cytoplasm (point ★) in SCC cells (Figure 4) are shown in Figure 5. The lower spectra indicate the 800–1800 cm^−1^ region, and the left side spectra are an expanded image of the 1650 cm^−1^ region. In Figure 5, the blue line is the spectrum of the cytoplasm (point ★) and the green line is the spectrum of the nuclei (point ▲) in the SCC cells. The peak at 1646 cm^−1^ in the cytoplasm is found to shift to 1650 cm^−1^ in the nucleus. The sharp peaks at 1650 cm^−1^ and 1436 cm^−1^ were confirmed in both areas of the cytoplasm and the nucleus.

### 2.3. Raman Mapping Images of SCC Cells

**Figure 6:**
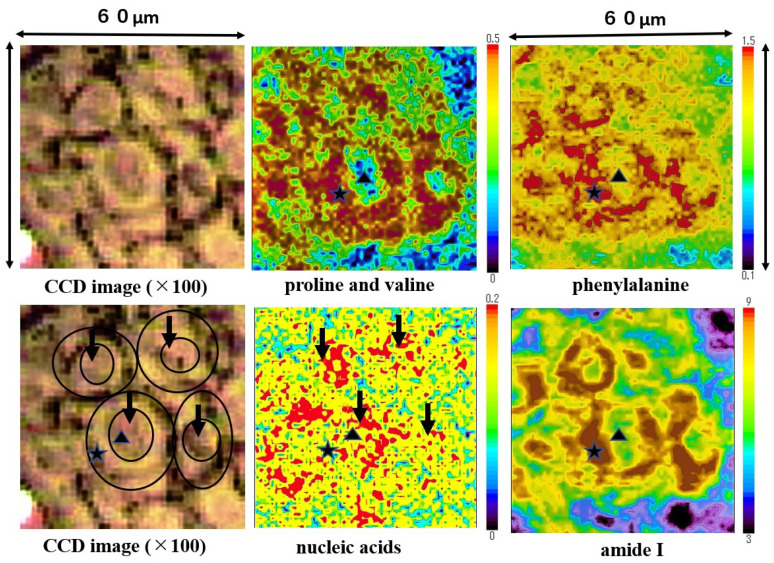
The mapping images proline and valine, phenylalanine, nucleic acids, and Amide I in the four-sided area of Figure 4 are shown. Proline and valine, phenylalanine, and amide I were detected mainly in the cytoplasm of SCC cells. In each image ▲ and ★ indicate the nucleus and ★ the cytoplasm individually, position of ▲ and ★ are all same.

Mapping images of the proline and valine (935 cm^−1^), phenylalanine (1004 cm^−1^), nucleic acids (1223 cm^−1^), and amide I (1650 cm^−1^) are shown in Figure 6. Nucleic acids were concentrated in the nuclei area (four black arrows). Proline and valine, phenylalanine and amide I were concentrated in the cytoplasm area, and the three external cell forms at the lower and upper left side were shaped by the minute distribution of these components. Amide I was depicted in the SCC cell outline most clearly. Nucleic acids were distributed surrounded by the cytoplasmic components. The size of a single SCC cell is approximately 15~20 µm in diameter (size); meanwhile, the nucleus is 5~10 µm in diameter size. The outline of SCC cells and position of nucleus on these mapping images corresponds to the image from the CCD camera equipped with a Raman micro-spectroscope (left side). Mapping measurements were also performed on normal epithelium cells. However, we could not obtain clear mapping images of normal cells with a distribution of the above components.

### 2.4. Peak Position of Amide I in the SCC and Normal Areas, and in the Nuclei and Cytoplasm of SCC Cells

The peak position of amide I is at 1648.7 cm^−1^ within the SCC area (Group B) and at 1645.2 cm^−1^ within the normal area (Group A) (Table 1). The average peak position of the amide I band, in the malignant area, shifted to a higher frequency compared with that of amide I in the normal area. In the SCC cells, the amide I band shifted to the high frequency side of about 3.9 cm^−1^ in the nuclei area (Group C) compared to the cytoplasm area (Group D). Significant differences (*p* < 0.05) were observed in the changes in the Amide I peak position between the SCC area and normal area, and in the nucleus and cytoplasm of the SCC cells.

## 3. Discussion

In earlier reports of malignant tissue based on Raman spectroscopy, the main discussion concerns the difference in spectra between malignant and normal tissues [3,4,5,6,11,12,13,14,15,16]. Based on the present study, however, we can, for the first time, identify the SCC cells as a mapping image reflecting the distributions of proline and valine, phenylalanine, nucleic acids, and amide I in oral SCC cells. We analyzed the Raman spectra of oral SCC tissue without the application of any particular pretreatment reagent, which allowed us to negate the influence of having fixed the tumor tissues with formaldehyde. It is important to keep the protein secondary conformation changes as raw tissue, which can be achieved without fixing based on Fourier-transform infrared (FT-IR) microscopy [17]. Furthermore, it is advantageous to analyze the cryo-sectioning sample containing the lipid components, which will be lost as a result of the de-paraffinization process with MeOH lines. In other studies, most of the samples have been treated with formaldehyde [6,18]. It has been pointed out that application of formaldehyde solution to the samples might cause significant changes in the Raman spectra of amino acid regions associated with proteins [19]. In addition, our analysis by Raman micro-spectroscopy was carried out by the contrasting of the H&E staining image.

It has been reported that the peak position at 1650~1655 cm^−1^ (amide I) in the normal area shifts to 1667~1668 cm^−1^ in the malignant region of the breasts and lungs carcinoma [20,21]. Likewise, our data indicated that the peak position of the amide I band shifted to a higher frequency in the malignant area compared with the normal area. Furthermore, the band of amide I within the nuclei of oral SCC cells was found to possess a higher frequency than the amide I band of the cytoplasm. Our data showed the significant (*p* < 0.05) difference in peak shift at a higher wave number of amide I (average 1648.7 cm^−1^) in the SCC region compared with the normal region (average 1645.2 cm^−1^). It is known that the change in the secondary protein structure is related to the peak shift in both malignant and normal tissues [20,21,22]. In normal tissue, the peaks at 1650~1655 cm^−1^ (amide I band) are related to the α-helix conformation of the protein. In short, it is reported that the peak shift to a higher frequency in the amide I band may be associated with an increase in the relative amounts of protein in the β-sheet or random coil conformation [20,21].

The comparison between peak position at amide I of the nuclei and those of the cytoplasm in oral SCC cells suggests that there will be larger changes in the secondary protein structure in the nucleic regions than in the cytoplasmatic regions. We successfully obtained the individual peak positions of nuclei and cytoplasm at amide I within the oral SCC cells for the first time. However, it was not possible to obtain a clear mapping image in the analysis of 960 μm square including normal epithelial and oral SCC region (Figure 3). It was considered that the increase of the distance between the measurement points (16 μm) had an effect to poor contrast image if the analysis region (960 μm square) was measured at 3600 points. But this measurement settings takes a long time to perform mapping analysis. Therefore, it is necessary to improve the long measurement time. Also, a clear image of any components could not be obtained by analysis of normal epithelial cell even if the morphology of normal cells could be confirmed with CCD camera. The distribution of amide I in normal tissues (Figure 3, region B) is more uniform than that in cancer tissues (Figure 3, region A), which may be the reason why imaging of normal cells was difficult.

The imaging reflects the delicate distribution of components (proline and valine, phenylalanine, nucleic acids, and amide I) in oral SCC cells. Nucleic acids are abundant in the nuclei region of SCC cells. Proline and valine and phenylalanine and amide I components are mainly distributed throughout the cytoplasm area in the SCC cells. The image of these components (proline and valine, phenylalanine, and amide I shaped as a ring) in SCC cells can be distinguished between the spectra of nuclei and these of cytoplasm. In short, we were able to obtain directly significant amounts of important information about SCC cells using the approach and techniques described herein. Imaging of SCC cells with Raman micro-spectroscope technique indicated that this new method can be useful for analyzing the detailed distribution of various components (amide I, nucleic acids, and so on) in SCC cells using common frozen sections. This study will be very important in the future for use in spectroscopic analysis and diagnosis of tumor tissue.

## 4. Materials and Methods

### 4.1. Preparation of the Samples

Fresh biopsy specimens were collected during routine surgical procedures from five patients, and these specimens were embedded immediately in Optimal Cutting Temperature (O.C.T.) Compound (TissueTeck, 4583 type, SAKURA Finetechnical Co., Ltd., Tokyo, Japan). After being instantaneously frozen with liquid nitrogen, serial transverse sections were prepared at 10-μm thickness with a cryostat instrument (CM1950-OUVVM type, Leica, Germany). One sample section was set onto a slide glass covered with gold (JASCO Ltd., Co, Tokyo, Japan) to avoid spectrum enhancement for a Raman micro-spectroscope (Microscopic Raman System, NRS-1000 MTS type, JASCO, Tokyo, Japan). The other sample section was mounted onto a slide glass, fixed in 10% formalin, and stained with hematoxylin and eosin (H&E). Ten sample sets (one side for Raman analysis, the other for H&E staining) were prepared from the five patients with oral SCC. The tissue of two patients with oral benign tumor resection was used as a normal sample. These samples were classified by a pathologist as either normal regions or oral SCC regions. The study was approved by the Ethical Committee of Fukui General Hospital, and all patients gave their written informed consent.

### 4.2. Instruments: Raman Micro-Spectroscope

Raman spectra were measured with a Raman micro-spectroscope (Microscopic Raman System, NRS-1000 MTS type, JASCO, Tokyo, Japan). The excitation wavelength was 785 nm, and the laser spot size was 4 μm. The central frequency was set at 1400 cm^−1^, and the laser power was 12.0 mW. The aperture size was 200 μm, the slit width 300 μm, and the objective lens had a 100× magnification. The diffraction grating of the Raman spectrometer was 1200/mm. The slide glass was horizontally set up on a measurement stage. The Raman spectra were recorded at room temperature. Raman frequencies were calibrated with the spectra of polypropylene to obtain accurate spectra before measurement. All Raman spectra were obtained with a 5 s exposure time, and the measurements were performed twice for each measurement point. All spectra were processed for smoothing by Means Movement (convolution width: 5). Raman spectra were corrected by subtracting a liner baseline between 800 cm^−1^ and 1800 cm^−1^. Smoothing and baseline collection were performed with the same settings for individual spectrum of the 3600 measurement points obtained in a mapping analysis. The peak positions and peak areas were analyzed after this process. The Raman peak area of these components was calculated as the inside area of individual at peaks 935 cm^−1^ (proline and valine), 1004 cm^−1^ (phenylalanine), 1223 cm^−1^ (nucleic acids), and 1650 cm^−1^ (amide I). For example, the peak area of amide I at 1645 cm^−1^ was calculated as at the range areas from 1640 cm^−1^ to 1660 cm^−1^.

### 4.3. Mapping Measurements of Oral SCC Tissues and Cells

Mapping measurements were performed with a lattice in the four-sided area automatically, and this area was measured minutely at 60 × 60 (total 3600) points (Figure 3 and Figure 6). The point-to-point distance was set to 16 μm (Figure 3) or 1 μm (Figure 6). The colors of the mapping image indicate that the differences in these component (proline and valine, phenylalanine, nucleic acids and amide I) quantities (on a descending scale in linear quantity: red > yellow > green > blue). We demonstrated one case involving both normal and SCC regions (Figure 3) and SCC cells. We classified region A as SCC and region B as normal on the H&E staining image (Figure 1). The mapping measurement area of cancerous cells was adopted as the group of SCC cells with large nuclei (Figure 4). Arrows ‘a’ show the large nuclei. These individual nuclei are surrounded by a pale cytoplasm ‘b’ and a distinct cell wall.

### 4.4. Analyses of the PeakPpositions of Amide I in Oral SCC Tissues and Cells

Peak position at amide I (1655 cm^−1^) was calculated as the mean value of individual peak positions at total 300 points in the normal regions (two samples) and at total 1000 points in SCC regions (ten samples), respectively (Table 1). For example, 100 measurement points in the region A on Figure 1 were defined as the spectrum of the cancer tissue, and 100 measurement points in the region B were defined as the spectrum of the normal tissue. Likewise, the peak position of amide I in the cytoplasm and the nucleus is the mean value of the peak position at 150 points in each area of SCC cells (two samples). We selected as the spectrum of the cytoplasm area (★), which contained a lot of proline and valine, phenylalanine, amide I, and, as the spectrum of the nuclei area (▲), which contained a lot of nucleic acids, as shown in Figure 6. Each mapping images in Figure 6 are composed of data at 3600 measurement points. Therefore, the position of ★ (22: x axis, 22: y axis) and ▲ (31: x axis, 25: y axis) indicates the same measurement points on each mapping image. Specifically, the point (31: x axis, 25: y axis (▲)) was chosen as the nucleic area and the point (22: x axis, 22: y axis (★)) was chosen as the cytoplasm area (Figure 6). In the same manner, 150 nuclei and cytoplasmic spectra were picked up. 

## Figures and Tables

**Table 1:** Peak position of amide I in the SCC and normal areas, and in the nuclei and cytoplasm of SCC cells. Sample numbers and measurement points are indicated.

		Average of Peak Position	SD	Welch’s Test
Group A	Normal sample 1 (*n* = 100 points)	1645.2 cm^−1^	1.14	*
	Normal sample 2–3 (*n* = 200 points)			
Group B	SCC sample 1–2 (*n* = 200 points) patient 1	1648.7 cm^−1^	1.49	*
	SCC sample 3–4 (*n* = 200 points) patient 2			
	SCC sample 5–7 (*n* = 300 points) patient 3			
	SCC sample 8–9 (*n* = 200 points) patient 4			
	SCC sample 10 (*n* = 100 points) patient 5			
Group C	Nuclei SCC sample 1–2 (*n* = 100 points) patient 1	1649.3 cm^−1^	1.57	*
	Nuclei SCC sample 3 (*n* = 50 points) patient 2			
Group D	Cytoplasm SCC sample 1–2 (*n* = 100 points) patient 1	1645.4 cm^−1^	1.18	*
	Cytoplasm SCC sample 3 (*n* = 50 points) patient 2			

* *p* < 0.05.

## Data Availability

Not applicable.

## Sample Availability

Not available.

## References

[1] Shiboski C.H., Shiboski S.C., Silverman S. (2000). Trends in oral cancer rates in the United States, 1973–1996. Community Dent. Oral Epidemiol..

[2] Long D.A. (2002). The Raman Effect: A Unified Treatment of the Theory of Raman Scattering by Molecules.

[3] Bourbousson M., Soomro I., Baldwin D., Notingher I. (2019). Ex vivo Raman spectroscopy mapping of lung tissue: Label-free molecular characterization of nontumorous and cancerous tissues. J. Med. Imaging.

[4] Ramos I.R., Malkin A., Lyng F.M. (2015). Current advances in the application of raman spectroscopy for molecular diagnosis of cervical cancer. Biomed. Res. Int..

[5] Zhang X., Yu F., Li J., Song D., Li H., Wang K., He Q., Wang S. (2019). Investigation on the Cancer Invasion and Metastasis of Skin Squamous Cell Carcinoma by Raman Spectroscopy. Molecules.

[6] Kaminaka S., Yamazaki H., Ito T., Kohda E., Hamaguchi H.-O. (2001). Near-infrared Raman spectroscopy of human lung tissues: Possibility of molecular-level cancer diagnosis. J. Raman Spectrosc..

[7] Paul T., Bruno P., Jean V., Bernard B., Luc M. (2000). A Raman spectroscopic investigation of dentin and enamel structures modified by lactic acid. Caries. Res..

[8] Tsuda H., Ruben J., Arends J. (1996). Raman spectra of human dentin mineral. Eur. J. Oral Sci..

[9] Kinoshita H., Miyoshi N., Miyoshi K., Ogawa T., Ogasawara T., Kitagawa Y., Itoh H., Sano K. (2008). Phosphate and amide III mapping in sialoliths with Raman microspectroscopy. J. Raman Spectrosc..

[10] Kinoshita H., Miyoshi N., Fukunaga Y., Ogawa T., Ogasawara T., Sano K. (2008). Functional mapping of carious enamel in human teeth with Raman microspectroscopy. J. Raman Spectrosc..

[11] Mahadevan-Jansen A., Richards-Kortum R. (1996). Raman spectroscopy for the detection of cancers and precancers. J. Biomed. Opt..

[12] Utzinger U.R.S., Heintzelman D.L., Mahadevan-Jansen A., Malpica A., Follen M., Richards-Kortum R. (2001). Near-Infrared Raman Spectroscopy for in vivo Detection of Cervical Precancers. Appl. Spectrosc..

[13] Jeng M.-J., Sharma M., Sharma L., Chao T.-Y., Huang S.-F., Chang L.-B., Wu S.-L., Chow L. (2019). Raman Spectroscopy Analysis for Optical Diagnosis of Oral Cancer Detection. J. Clin. Med..

[14] Venkatakrishna K., Kurien J., Pai K.M., Valiathan M., Kumar N.N., Murali Krishna C., Ullas G., Kartha V.B. (2001). Optical pathology of oral tissue: A Raman spectroscopy diagnostic method. Curr. Sci..

[15] Yang Y., Liu C.H., Savage H.E., Schantz S.P., Alfano R.R. (1998). Optical fluorescence and Raman biopsy of squamous cell carcinoma from the head and neck. Proc. SPIE.

[16] Feld M.S., Manoharan R., Salenius J., Orenstein-Carndona J., Roemer T.J., Brennan J.F., Dasari R.R., Wang Y. (1995). Detection and characterization of human tissue lesions with near-infrared Raman spectroscopy. Proc. SPIE.

[17] Yamada T., Miyoshi N., Ogawa T., Akao K., Fukuda M., Ogasawara T., Kitagawa Y., Sano K. (2002). Observation of molecular changes of a necrotic tissue from a murine carcinoma by Fourier-transform infrared microspectroscopy. Clin. Cancer Res..

[18] Krishna C.M., Sockalingum G.D., Kurien J., Rao L., Venteo L., Pluot M., Manfait M., Kartha V.B. (2004). Micro-Raman Spectroscopy for Optical Pathology of Oral Squamous Cell Carcinoma. Appl. Spectrosc..

[19] Huang Z., McWilliams A., Lam S., English J., McLean D.I., Lui H., Zeng H. (2003). Effect of formalin fixation on the near-infrared Raman spectroscopy of normal and cancerous human bronchial tissues. Int. J. Oncol..

[20] Huang Z., McWilliams A., Lui H., McLean D.I., Lam S., Zeng H. (2003). Near-infrared Raman spectroscopy for optical diagnosis of lung cancer. Int. J. Cancer.

[21] Manoharan R., Shafer K., Perelman L., Wu J., Chen K., Deinum G., Fitzmaurice M., Myles J., Crowe J., Dasari R.R. (1998). Raman spectroscopy and fluorescence photon migration for breast cancer diagnosis and imaging. Photochem. Photobiol..

[22] Stone N., Stavroulaki P., Kendall C., Birchall M., Barr H. (2000). Raman Spectroscopy for Early Detection of Laryngeal Malignancy: Preliminary Results. Laryngoscope.

